# Geopolymerization of Kaolin Clay with Hemp Fibers for Sustainable Soil Stabilization

**DOI:** 10.3390/polym17233216

**Published:** 2025-12-02

**Authors:** Bilge Aksu Alcan, Halil Oğuzhan Kara, Mehmet Uğur Yılmazoğlu

**Affiliations:** 1 Department of Civil Engineering, Kafkas University, Kars 36100, Türkiye; 2 Department of Civil Engineering, Kastamonu University, Kastamonu 37150, Türkiye; hokara@kastamonu.edu.tr (H.O.K.); myilmazoglu@kastamonu.edu.tr (M.U.Y.)

**Keywords:** stabilization of soil, hemp fiber, geopolymer, freeze–thaw cycle, carbon index

## Abstract

In this study, the aim was to improve the mechanical and durability properties of kaolin clay (KC)-based soil by stabilizing it with geopolymer and natural fiber. In the production of the geopolymer, rice husk ash (RHA) was used as a binder, sodium metasilicate (SMS) as an activator, and another hemp fiber (HF)was used for soil stabilization. Within the scope of the presented study, RHA and SMS were used at three different rates (5%, 7.5%, and 10%), while HF was used in six different volumes (0.5%, 1%, 1.5%, 2%, 2.5%, and 3%) and two different lengths (6 and 12 mm). The study also examined how much water was in the combinations, which was measured at the optimum level and at −5, +5, and +10 compared to the optimum level. The unconfined compressive strength (UCS) was used to check the mechanical qualities of the test specimens and 5- and 10-cycle freeze–thaw (F-T) tests to check the durability properties. The test results indicated that the mixed formulation with 5% RHA, 10% SMS, 2.5% HF, and the optimum water content resulted in the best results for both the UCS and F-T tests. The SEM investigation for this mix found that the microstructural properties for the specimen were directly related to the dense gel phases and the strong fiber–matrix bonding. According to the carbon emissions (CO_2_-e) and carbon index (CI) analysis from the mix component analyses, it was found that the HF-strengthened geopolymer is a sustainable solution for soil stabilization. The optimum mixture achieved a UCS of 1202 kPa (4.5 times higher than untreated soil), while the strength losses after 10 freeze–thaw cycles were reduced to below 10% in optimized compositions. The carbon index (CI) decreased by up to 65%, demonstrating the strong sustainability benefits of the proposed system. The novelty of this study lies in the combined use of hemp fiber (HF) and rice husk ash (RHA)–sodium metasilicate (SMS)-based geopolymer for kaolin clay stabilization, which has not been comprehensively investigated in previous research. Unlike traditional studies focusing on either geopolymer or natural fiber reinforcement alone, this work simultaneously evaluates the mechanical performance, freeze–thaw durability, microstructural evolution, and carbon footprint to develop a fully sustainable soil improvement framework.

## 1. Introduction

A common problem associated with the branch of geotechnical engineering is the significant sensitivity exhibited by natural clays, especially with respect to moisture. Such soil materials exhibit undesired engineering properties, such as volumetric instability, poor strength, high compressibility, and time-dependent deformation. Notably, clays with a mineralistic character and those with higher plasticity indices, for example, kaolin, pose a threat to the geotechnical infrastructures, for instance, embankment roads, dam foundations, and soil foundations. The most common method employed for the stabilization of these soils is the application of stabilization techniques, which involve the use of chemical, physical, or biological alteration approaches. The classical approaches, however, use calcium-based binders, for example, cement and lime; these are expected to react with the earth, producing hydration and pozzolanic products [1,2]. However, the classical approaches have some associated deficiencies. First, the energy utilized in the production process for Portland cement and lime is very significant, and the process also creates high volumes of atmospheric CO_2_ emissions. It has been confirmed that the production process for cement alone is responsible for approximately 7% of the overall carbon emissions to the atmosphere [3]. In addition, the excessive usage of standard binders can lead to recurring microstructural defects, such as carbonation-related fissures and sulfate reactions [4]. Therefore, with the continuous quest for sustainable materials, there has been considerable interest in novel binders that include lower carbon emissions. The technology of the geopolymer is an example, and it has attained prominence within the disciplines of geotechnical engineering over the last few years due to its environmental advantages, as well as its technical strength.

Geopolymers (GP) are generally synthesized by the reaction between waste or naturally occurring materials rich with silicon (Si) and aluminum (Al) and highly alkaline activators. Based on the process occurring in the formation, geopolymers are also referred to, in the literature, simply as alkali-activated binders (AAB) [5,6]. The geopolymerization reactions between precursor (binder) materials and other activating agents result in the formation of three-dimensional amorphous aluminosilicate gel networks. The resulting gel differs from the conventional cement-based formation of the C-S-H. It is, in general, representative of the formation of N-A-S-H (sodium aluminosilicate hydrate) or C-A-S-H gel [7,8,9]. The major precursor materials utilized for GP production include fly ash (FA), ground granulated blast furnace slag (GGBFS), metakaolin, volcanic tuff, pumice, rice husk ash (RHA), palm oil ashes, and some varieties of natural clays (particularly kaolin). They are all economically beneficial, easily obtained, and often classified under the category of industrial waste, hence bringing significant benefits to the field of sustainability [10,11]. The use of agricultural wastes, especially RHA, is fundamental to the production of environmentally friendly binders. In addition, some studies presented in the literature suggest that natural minerals derived from clays, for example, kaolin, can also represent potential sources for aluminosilicates. Alkali activators that are utilized for the manufacturing process for GP include sodium hydroxide (NaOH), sodium metasilicate (Na_2_SiO_3_), and potassium hydroxide (KOH) [12,13,14].

The efficacy of geopolymer binder systems in soil applications extends beyond the mere enhancement of strength. A multitude of studies documented in the literature indicate that these systems markedly enhance various soil characteristics, including permeability, volume stability, resistance to freeze–thaw cycles, durability, and even chemical resilience. Nevertheless, a notable drawback associated with geopolymer-stabilized soils is their brittle mechanical nature. Specifically, cracks that may occur in soil matrices, exhibiting low tensile strength, can impede deformation capabilities under applied loads. To address this challenge, the incorporation of fiber reinforcement is recognized as a significant strategy [15]. By evenly distributing fibers throughout the soil matrix, the progression of cracks can be mitigated, load distribution can achieve greater isotropy, and the capacity for energy dissipation can be augmented. At this juncture, the implementation of eco-friendly organic fibers, such as hemp, coconut, and sisal, in soil stabilization presents both technical benefits and ecological advantages [16,17,18]. Hemp fiber (HF) emerges as a promising reinforcement material for improving the ductility and durability characteristics of soils, attributed to its properties, including high tensile strength, low density, alkali resistance, biodegradability, and cost-effectiveness. Furthermore, during its cultivation, hemp sequesters carbon dioxide (CO_2_) from the atmosphere, storing this carbon within its fibers and roots [19]. This distinctive property positions hemp as a negative carbon sink, thereby enhancing its significance. An array of studies has demonstrated that geopolymer-stabilized soils integrated with HF exhibit enhanced performance in terms of both strength and environmental resilience. Additionally, it has been highlighted that HF-reinforced soils inhibit crack propagation, strengthen microstructural integrity, and improve tensile characteristics [20,21].

The present study aims to develop an alternative perspective concerning the field of geotechnical engineering regarding sustainable approaches to soil improvement. The geopolymer system here is comprised of using agricultural waste, namely rice husk ash (RHA), to serve as an aluminosilicate source, offering an environmentally more sustainable, renewable, and economical binding agent. The addition of hemp fibers (HF) to the scope of the study greatly improves the soil matrix through the provision of ductility and durability, consequently reducing the brittleness inherent to conventional geopolymer systems. The study entails an assortment of rice husk ash (RHA) and sodium metasilicate proportions (% 0, 5, 7.5, and 10) in the manufacture of the geopolymer, with the addition of 6 and 12 mm hemp fibers, introduced at the range 0–1.5%. The parameter of water content has been methodically studied at four levels: ±%5 and +%10 with respect to the optimum water content. The aforementioned test variables were structured, following the Taguchi L_16_ design for an orthogonal design, with the mechanical performance quantified using the unconfined compressive strength (UCS) parameter. In contrast, the environmental robustness was assessed through the application of five and ten freeze–thaw cycles. Lastly, the environmental footprint assessment simply came through the use of sustainability indicators, namely carbon footprint (kgCO_2_/kg) and carbon efficiency (CI).

### Research Significance

Original aspects of this research are the comprehensive analysis of soil stabilization with an agricultural waste-derived geopolymer, augmented with natural fiber reinforcement. The results are anticipated to formulate a novel framework for sustainable and high-performance solutions for soil enhancement. The presented study is not limited to technical contributions at the research laboratory scale; it also directly contributes to global objectives such as waste management and the development of low-carbon infrastructure.

## 2. Experimental Program

### 2.1. Materials

#### 2.1.1. Soil

**Figure 1:**
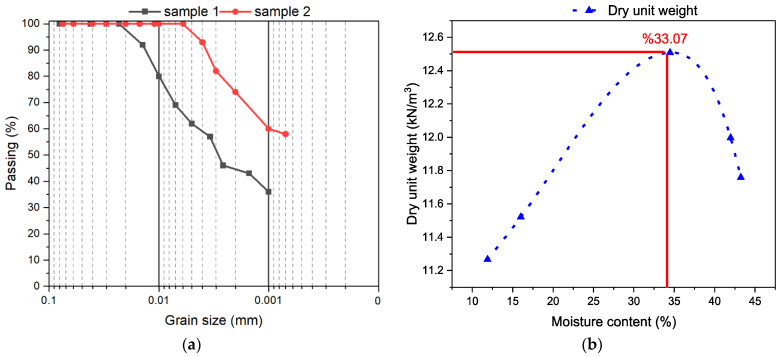
(**a**) Soil particle size distribution curve. (**b**) Proctor compaction curve.

**Figure 2:**
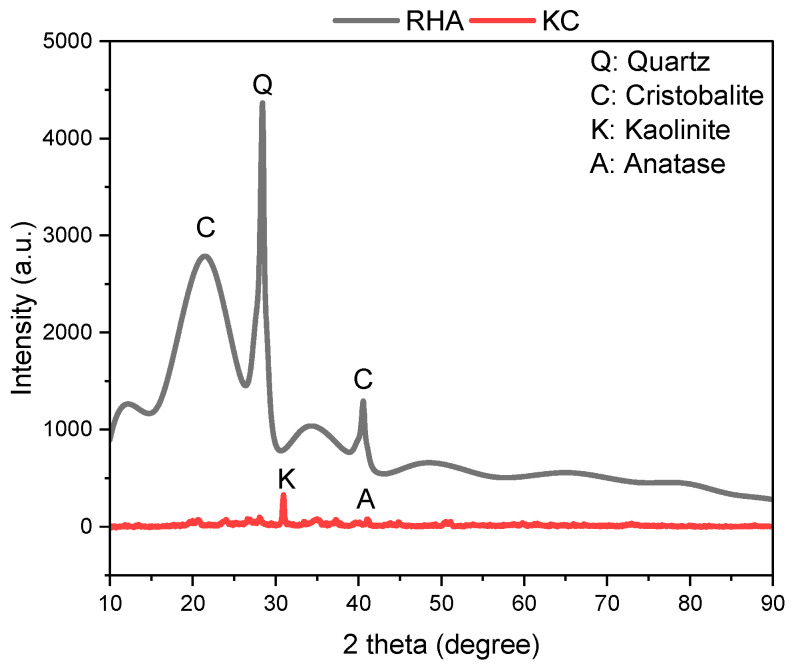
XRD pattern of RHA and KC.

The natural soil used in the present study is kaolin clay (KC) obtained from Kastamonu province in Turkey. This clay, which mainly consists of the mineral kaolinite (Al_2_Si_2_O_5_(OH)_4_), is formed by the weathering of granite and other igneous/volcanic rocks. The unit weight of the kaolin clay used in the study is 2.64 g/cm^3^. The gradation curve and optimum moisture content (OMC) 33.07% obtained from the Proctor test are presented in Figure 1. According to ASTM D2487 [22] and the Unified Soil Classification System (USCS), this kaolin falls into the low plasticity inorganic clay (CL) category, and the index properties of the soil sample are given in Table 1. The chemical composition of the clay used in the experiments is presented in Table 2, and the XRD pattern of KC is shown in Figure 2.

#### 2.1.2. Geopolymer Components

The precursor material of the geopolymer produced for soil stabilization is rice husk ash (RHA). RHA is obtained by controlled burning of waste rice husks at approximately 700–800 °C, followed by either rapid or slow cooling. Containing a high amount (80–90%) of amorphous silica, RHA exhibits pozzolanic properties due to its high specific surface area [23,24]. The average particle size of the RHA used in this study is 10 μm, and its unit weight is 2.28 g/cm^3^. In the experimental study, it was added to kaolin clay at rates of 0%, 5%, 7.5%, and 10% by weight. The chemical composition of RHA is given in Table 2, and the XRD pattern is shown in Figure 2. As seen in the XRD pattern, it was determined that RHA contains a high amount of amorphous phase.

**Figure 3:**
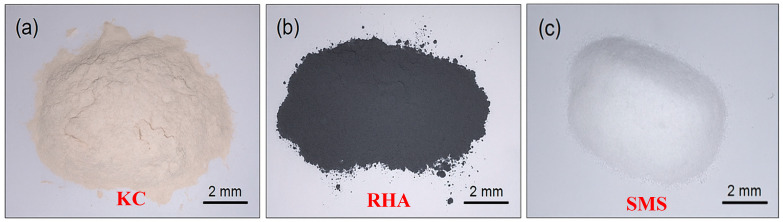
Raw materials used in experiments. (**a**) KC; (**b**) RHA; (**c**) SMS.

**Figure 4:**
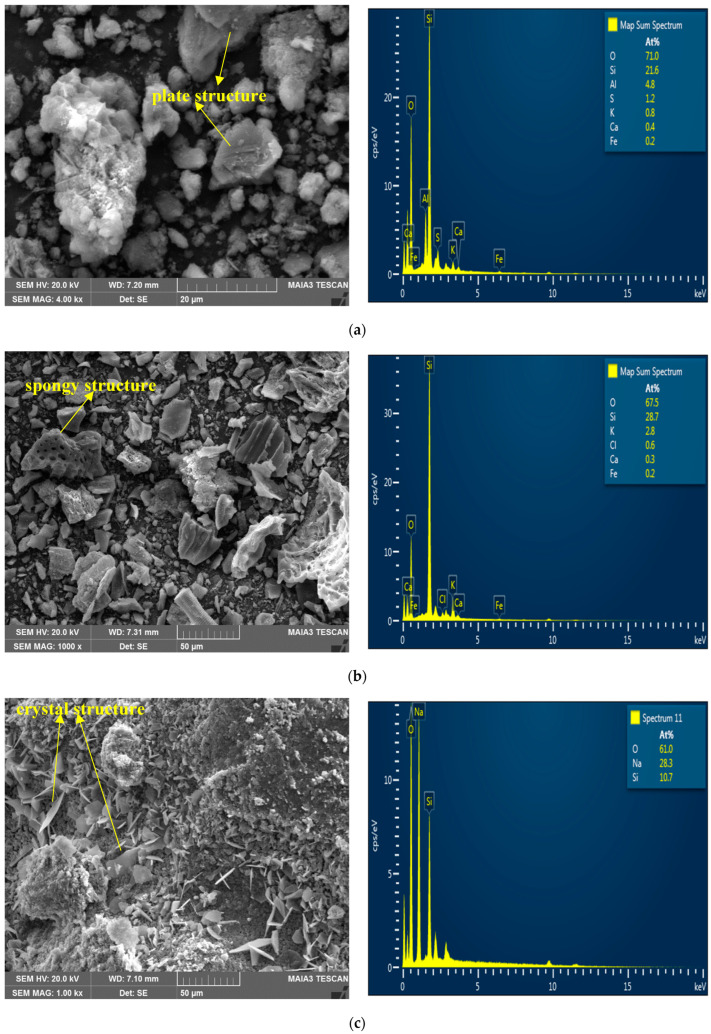
SEM and EDS analysis of materials used in geopolymer components: (**a**) KC; (**b**) RHA; (**c**) SMS.

Sodium metasilicate (SMS) was used to activate RHA in geopolymer production. SMS (Na_2_SiO_3_), also known as water glass, is an inorganic compound in powder form. The use of SMS as an alkaline activator increases the solubility of Si and Al content in the soil, thereby accelerating the formation of geopolymer binders [25,26]. The SMS used in this study has a specific gravity of 2.63 g/cm^3^ and a molecular weight of 122.06 g/mol. In addition, the pH value of SMS in a 1% solution is 12.5, and its melting point is approximately 1088 °C. Raw materials (KC, RHA, and SMS) used in the scope of this study were procured from local companies in Türkiye. The SMS additive was used at rates of 0%, 5%, 7.5%, and 10% by KC weight. Potable water was used to activate the alkalis. The water content used in the samples constitutes another parameter of the study. Within the scope of the study, the water content was determined at four levels based on the optimum moisture content (%OMC) specified for the soil: %OMC−5, %OMC, %OMC+5, and %OMC+10. In the study, three types of raw materials were used: kaolin clay (KC), rice husk ash (RHA), and sodium metasilicate (SMS), which are shown in Figure 3. SEM images and EDS analyses of the materials used in the experiments are presented in Figure 4. In Figure 4a, the SEM analyses of KC are shown: the presence of layer-like structures indicates the existence of kaolinite, and these results are in parallel with the XRD results presented in Figure 2. The EDS results of KC confirm both the XRD and the chemical components. In Figure 4b, SEM images of RHA, highly porous and irregular spongy structures, are identified, and the EDS results are consistent with the chemical components presented in Table 2. When examining Figure 4c, SEM images of SMS, a dense crystalline structure is observed, while the EDS results show the three main elements that make up this component.

#### 2.1.3. Hemp Fiber

**Figure 5:**
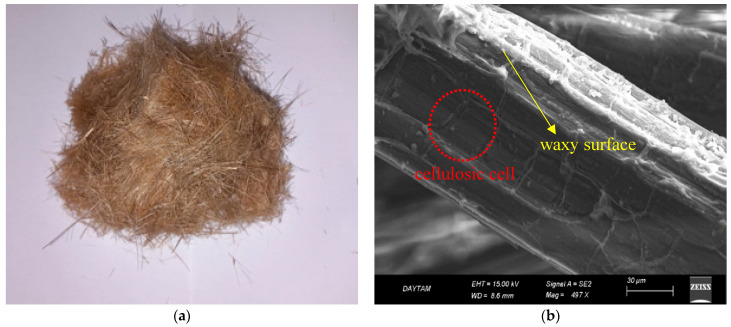
(**a**) Physical appearance of HF. (**b**) SEM image of HF.

In the current investigation, hemp fiber (HF) was selected for soil stabilization. Hemp fibers, which are sourced from the Cannabis sativa plant, are categorized as natural fibers. HF that is derived from organic waste is particularly distinguished by its robustness, stiffness, and lightweight characteristics; it does not contribute to environmental pollution and demonstrates resistance to biological decay. Another reason for the preference for HF in this research is its ability to sequester carbon dioxide (CO_2_). The carbon-negative nature of HF significantly bolsters sustainability efforts by reducing greenhouse gas emissions in applications related to soil stabilization and construction while simultaneously offering ecological advantages. In the experiments conducted, HF was prepared in two different lengths, 6 and 12 mm. The unit volume weight of the hemp fiber used is 1.4 g/cm^3^, and it was added to the mixtures at 0%, 0.5%, 1%, and 1.5% by weight. Table 3 displays the mechanical and physical properties of HF, whereas Figure 5 illustrates the overall appearance of HF along with its Scanning Electron Microscope (SEM) image.

### 2.2. Experimental Design

In the current study, an experimental design table was created, as presented in Table 4, using MINITAB software (Version 22.1) and the Taguchi experimental design methodology to optimize multiple parameters and levels simultaneously. An L16 orthogonal array was used for these parameters, and a total of 16 different experimental combinations were created, as seen in Table 5. The experimental combinations were prepared following the principle of randomness to enhance the reliability of the results [29]. In addition, to determine the extent to which each parameter considered affects the experimental results, a statistical analysis method called ANOVA (Analysis of Variance) was applied. The RHA and SMS dosages, the length and volume of HF, and the amount of water determined in the study were carefully selected based on both a literature review and preliminary experiments.

The Taguchi method used in the study employs an analysis based on the principle of converting experimental results into a signal-to-noise (S/N) ratio. In this method, S and N represent the signal factor and noise factor, respectively. This study focused on five different factors, each with four different levels. Since the aim is to maximize the unconfined compressive strength depending on the factors and levels, the “the larger, the better” criterion was adopted for the calculation of the S/N ratio. When evaluating freeze–thaw data, the “the smaller, the better” criterion was used in assessing the strength and mass loss ratios. In the analyses conducted, Equation (1) was used to determine the S/N ratio. The MSD (mean squared deviation) values are calculated using Equations (2) and (3) for the conditions where the target value is at its maximum and minimum, respectively. Here, n denotes the number of observations, and *y_i_* represents the *i*-th observation. In this study, maximization and minimization functions were used.

S/N = −10log_10_ [MSD] (1)

(2) $${MSD}_{\max}\;=\;\frac{1}{n}\;\sum_{1}^{n}\frac{1}{y_{i}^{2}}$$

(3) $${MSD}_{\min}\;=\;\frac{1}{n}\;\sum_{1}^{n}y_{i}^{2}$$

### 2.3. Preparation of Test Specimens

According to the mixture ratios presented in Table 5, the components of the 16 groups were weighed with precision. The solid components—kaolin clay (KC), rice husk ash (RHA), and sodium metasilicate (SMS)—were mixed in a Hobart mixer for 5 min to ensure homogeneity. Then, water was added to the mixtures according to the predetermined amounts for each group, and mixing continued for another 5 min. The freshly produced mixtures were placed into cylindrical molds with a diameter of 50 mm and a height of 100 mm in two layers. After the mixtures were placed into the molds, their weights were measured with precision. The test specimens produced were kept in the molds for 1 day, after which the molds were removed, and the specimens were placed in plastic bags to protect them from humidity. All the samples were cured in laboratory conditions (at room temperature) for 7, 28, and 90 days. Heat curing is a parameter that positively affects the rate of geopolymerization. Although it is known that applying heat curing to geopolymers increases the rate of gel product formation, in this study, heat curing was not applied to create an environment more similar to field conditions [30,31,32]. For the accuracy of the results, three samples were produced in each group, and their average was taken. A total of 240 test specimens were produced throughout the entire study.

### 2.4. Testing Procedure

#### 2.4.1. Unconfined Compressive Strength

As presented in Table 5, the unconfined compressive strength (UCS) test was applied to 16 different mixtures in accordance with ASTM D1633–17 [33]. For each group, the results were determined by taking the maximum strength obtained from the UCS test and averaging the stress–strain curves of the three mixtures produced. For this test, samples with a height of 100 mm and a diameter of 5 mm (L/D = 2) were used. The loading rate of the device used for the UCS test is 0.8 mm/min, and it has the capability to measure the axial deformation during the experiment. The energy absorption values of the samples were calculated by determining the area under the stress–strain curve obtained from the UCS test.

#### 2.4.2. Freeze–Thaw

In light of this investigation, an analysis was carried out to determine the environmental resistance of the mixtures through the process, where the stabilized soil samples were subjected to freeze–thaw (F-T) tests according to the ASTM D560 [34] standard. In this process, the stabilized soil samples, with a curing time of 28 days, were subjected to F-T cycles under the requirements for the F-T test, where the soil samples were frozen at −23 °C for 24 h and then thawed at ambient temperature for 24 h, under the test conditions of 98% relative humidity. Two different numbers of freeze–thaw cycles, namely 5 and 10 cycles, were applied to the stabilized soil samples. The stabilized soil samples subjected to the F-T cycles were measured for the unconfined compressive strength, energy absorption capacity, and mass losses.

#### 2.4.3. Microstructure Analysis

For this analysis, ZEISS scanning electron microscope (SEM) at the MERLAB facility of Kastamonu University and the DAYTAM facility at Atatürk University were used. Microstructural tests were performed for a selection of the test materials to examine the soil stabilization process with respect to time and environmental changes. In the SEM analyses, the formation of geopolymerization, fiber distribution, and pore structure in the test samples were examined.

#### 2.4.4. Carbon Footprint and Sustainability Analyses

In the presented study, the carbon footprint value of each stabilized soil sample was calculated. During these calculations, the CO_2_ emission (CO_2_-e) values of the binder (RHA), alkaline activator (SMS), hemp fiber (HF), and water used in each sample were taken into account. In addition, the carbon emission values resulting from the production and transportation processes of each component were also considered in these calculations [35]. The CO_2_-e value of each material used for soil stabilization is presented in Table 6. It was previously mentioned that the test samples were cured under ambient conditions. Considering that heat curing increases the CO_2_-e values, it is evident that ambient curing is a more sustainable and environmentally friendly method. This is another reason why ambient curing was preferred for the test samples. Another value calculated in the presented study is the carbon index (CI). As seen in Equation (4), this value is calculated as the ratio of the total measured carbon amount in the mixture (kg-CO_2_/ton) to the 28-day UCS (MPa) value. A decrease in the CI value indicates an improvement in the sustainability performance of the mixture.

(4) $$CI=\frac{Total\;{CO}_{2}\text{-}e}{{UCS}_{28day}}$$

## 3. Results and Discussion

In this section, the mechanical and environmental performance results of kaolin clay (KC) samples stabilized with geopolymer are presented; the findings obtained are evaluated and interpreted in line with the Taguchi experimental design. Unconfined compressive strength (UCS) test results, the effects of freeze–thaw cycles, carbon footprint and carbon efficiency, and microstructure analyses are discussed in detail under separate subheadings.

### 3.1. Unconfined Compressive Strength (UCS) Results

#### 3.1.1. The Effect of Curing Periods on UCS

**Figure 6:**
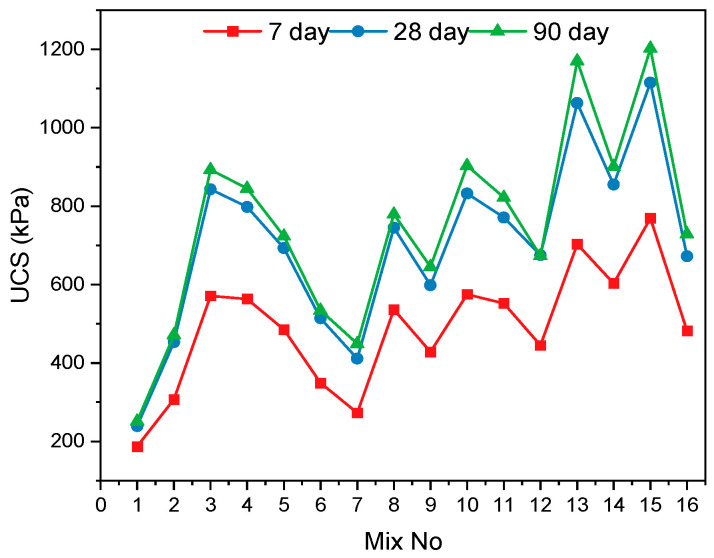
UCS values of mixtures according to different curing periods.

In this study, the UCS test results of geopolymer-stabilized KC samples at 7, 28, and 90 days are presented in Figure 6. Sixteen mixture samples with different additive combinations (Mix 1–16) were evaluated, and it was observed that the UCS values increased significantly with longer curing durations in all mixtures. Upon evaluating the results, it was found that, compared to the UCS values of the unstabilized soil sample (Mix 1) at different curing durations, the UCS value in the samples stabilized with geopolymer and added HF increased approximately 1.4 to 4.5 times. This implies that the addition of geopolymer and hemp fiber strengthens the mechanical character of soils. With respect to the curing time, it is clear that the reactions leading to geopolymerization develop gradually, consequently reinforcing the internal framework of the soil. While the strengths in the first 7 days generally remain low, a significant increase was observed between the 28- and 90-day strengths. Especially in mixtures 13, 14, and 15, the strength values obtained at the end of 28 and 90 days reveal that synergistic effects occurred between the components forming the mixtures and that suitable combinations can optimize mechanical performance. This general trend demonstrates that the effects of time and mixture compositions are of critical importance in the stabilization process [41,42].

**Figure 7:**
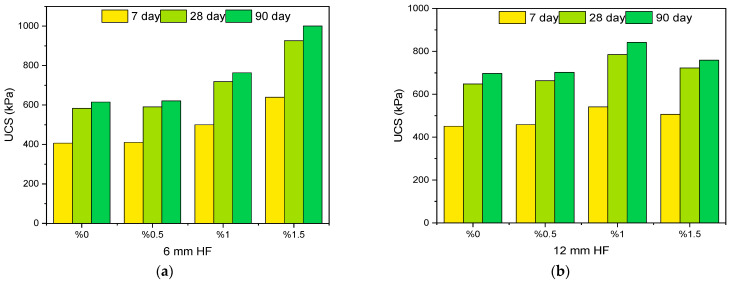

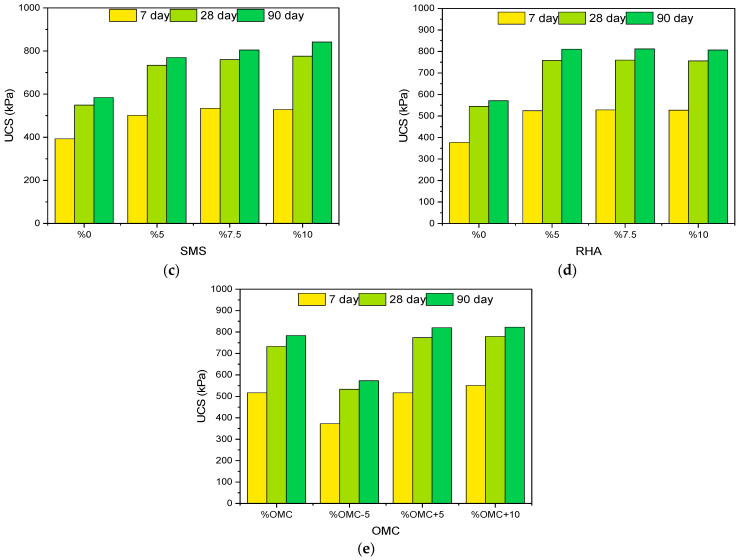
UCS values of test samples according to different parameters. (**a**) 6 mm HF effect; (**b**) 12 mm HF effect; (**c**) SMS effect; (**d**) RHA effect; (**e**) OMC effect.

The changes in 7, 28, and 90-day UCS results according to the varying levels of each experimental parameter are presented in Figure 7.

#### 3.1.2. Effect of Hemp Fiber (HF) on UCS

Figure 7a shows that with the increase in the proportion of hemp fiber with a length of 6 mm, there is a noticeable increase in UCS values at all curing durations. The lowest strength was observed in the samples without additives (0% HF), while the highest strength was obtained in samples containing 1.5% fiber. For example, the UCS value of the soil sample with 1.5% fiber is approximately 1.5 times higher than that of the sample without fiber. The HF added to the mixtures increased the soil–fiber adhesion, enabling the fibers to function as micro-reinforcement within the soil matrix. This effect limited the tensile cracking of the samples and increased their load-bearing capacity. Similarly, the literature reports that the addition of plant fibers increases the plastic deformation resistance and improves the strength of soils [43,44]. In Figure 7b, it can be seen that increasing the contribution of a 12 mm long hemp fiber up to a ratio of 1% has a positive effect on UCS. Notably, at 1% fiber content, the strength reaches its highest level at both 28 and 90 days of curing. At this point, longer fibers provide more effective tensile strength in the soil matrix, and the bond strength between the fiber and the matrix is high. However, when the fiber content is increased to 1.5%, a decrease in UCS values is observed. This situation can be attributed to fiber balling and reduced workability due to the excessive use of long fibers. This observation is consistent with the literature suggesting that when the fiber ratio exceeds a certain optimum level, the soil–fiber interaction may become disadvantageous and have a negative effect on strength [45,46,47]. As a result, the addition of HF in soil stabilization significantly increases the unconfined compressive strength of KC. HF improves UCS by acting as a crack-bridging reinforcement. The rough surface of the fibers enhances interfacial bonding, enabling them to carry tensile stresses and delay crack propagation. This mechanism increases the ductility of the matrix and improves the load distribution, thereby enhancing the compressive capacity. While increasing the fiber ratio to 1.5% can be beneficial in some cases, a decrease in strength may also be observed due to the difficulty in achieving a homogeneous mixture. Therefore, determining the optimum fiber length and ratio is of critical importance for mechanical performance improvement.

#### 3.1.3. Effect of Sodium Metasilicate (SMS) on UCS

In Figure 7c, it is found that an increase in the SMS ratio from 0% to 7.5% results in a drastic rise in UCS values. Such an increase is largely found at the 28-day and 90-day curing stages. It is found that the process of geopolymerization occurs at a lower rate at earlier stages of curing. The mix with 7.5% SMS again showed the highest value of strength; however, if the ratio of SMS is increased further to 10%, the trend of increasing UCS values tended to shift downwards. For instance, the 0% SMS-containing control mix showed a value of 585 kPa at 90 days, whereas the 7.5% and 10% SMS-containing mixes showed 805 kPa and 835 kPa, respectively. Such a trend is the result of the addition of SMS to the process of alkali activation, the progress of the pozzolanic reactions, and the increase in the production of binding products, primarily N–A–S–H and C–A–S–H gels [48]. These gels form binding network structures between soil particles, thereby improving the strength. However, when the amount of activator increases excessively, it can lead to a disruption of the ionic balance in the system, deterioration of the matrix microstructure due to excess alkalinity, and irregularity in the formation of reaction products, all of which may cause a loss of strength. This observation likewise supports studies reporting that the strength of binders containing sodium silicate may decrease after exceeding the optimum dosage [49,50,51]. As a result, the addition of SMS significantly increases the mechanical strength of kaolin clay (KC), mainly when used in the 5–7.5% range. However, the use of a 10% addition caused a disruption in the chemical balance and limited the increase in strength. Therefore, the optimum ratio of SMS should be carefully determined by considering the soil type and other additives.

#### 3.1.4. Effect of Rice Husk Ash (RHA) on UCS

As can be seen in Figure 7d, increasing the RHA addition rate from 0% to 7.5% resulted in an increase in UCS values. Especially in the 28- and 90-day curing periods, the mixture containing 7.5% RHA showed the highest strength values in all time frames. However, at an addition rate of 10%, the UCS values showed a slight decrease. The control sample without RHA showed a strength of 585 kPa at the end of the 90-day curing period. In mixtures containing 5% RHA, this value increased by approximately 1.4 times, and in those containing 7.5% and 10% RHA, this increase reached 1.5 and 1.44 times, respectively. The high amorphous silica content in the RHA reacts with the water and activator in the mixture to form N-A-S-H (sodium–alumina–silicate–hydrate) gel. This gel improves the microstructure of the matrix, thereby increasing the strength. This pozzolanic component, in particular, improved the binding capacity of the clay and increased the strength by reducing the void ratio [52,53,54]. As is evident from the result at the 10% significance level, this diminution is due to the presence of excess silica in the form of an unreacted component within the mixture, leading to the formation of microcracks and weakness points. In agreement with the result for the present study, it has been found through earlier studies that excessive silica content has the potential to weaken binding phases and diminish workability through water absorption [55]. Therefore, the adoption of RHA, especially within the range of 5–7.5%, significantly strengthens the unconfined compressive strength of kaolin clay. The higher silica content allows for the proper formation of binding gels, leading to strengthened soil matrix density, hence the overall improvement. However, additions above or at the 10% limit are subject to critical evaluation, since exceeding this limit will cause shifting of the reaction equilibrium and the formation of a non-uniform binder form.

#### 3.1.5. Effect of Optimum Moisture Content (OMC) on UCS

In Figure 7e, the results for the unconfined compressive strength (UCS) were evaluated through the variation of the water content at four different levels: %OMC, %OMC−5, %OMC+5, and %OMC+10. In general, the samples formulated at the %OMC+5 grade showed the best strength readings at both the 28-day and 90-day curing periods. Specifically, with %OMC+5 water content, the maximum UCS reading of 1202 kPa was achieved after 90 days of curing. This indicates that when the water content is slightly above the optimum value, the soil matrix contains enough moisture to support chemical reactions. Specifically, the presence of adequate water is critical for the formation of the impermeable geopolymer gel, particularly in alkali-activated binders [56,57]. The water content at the %OMC+5 grade successfully balanced both the precursor and activator within the mix, consequently improving the strength development. However, at the %OMC+10 grade, the increase in the UCS values tended to flatten. The above is due to the excess water, through the formation of voids within the microstructure, impeding the progression of strengths [58]. Conversely, for the %OMC−5 water content, the resulting UCS readings were remarkably suppressed at all ages. The above outcome is mainly attributed to the inhibited geopolymerization reactions due to the insufficient number of water volumes. Furthermore, under this water content (%OMC−5), HF may have exhibited an effect of absorbing the available water, further reducing the reactions. The literature indicates that less than the required amount of water hinders the formation of binding phases in geopolymers [59,60,61]. Based on the results of this study, the relationship between water content and UCS is not linear; deviations from the optimum point (whether too little or too much water) negatively affect the strength. The %OMC and %OMC+5 levels provided the most suitable environment for geopolymerization and fiber–matrix adhesion, along with additives such as RHA, SMS, and HF, to stabilize the kaolin clay.

#### 3.1.6. Statistical Analysis of UCS Results According to Parameters

**Figure 8:**
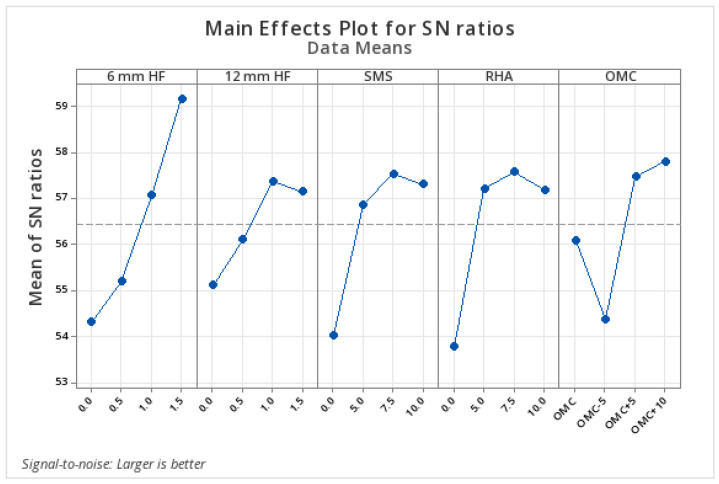
Twenty-eight-day unconfined compressive strength S/N chart.

In this study, the analysis of the average effects of the factors based on UCS data obtained after the 28-day curing period is presented in Table 7, and the Main Effects Plot chart regarding the signal/noise (S/N) ratios is presented in Figure 8. In the analysis conducted within the scope of the Taguchi experimental design, the contribution rate of the 6 mm long hemp fiber (HF) stood out as the most dominant parameter with a Delta value of 4.85. This was followed by rice husk ash (RHA), sodium metasilicate (SMS), optimum moisture content (OMC), and the addition of HF with a length of 12 mm, respectively.

The results of the variance analysis (ANOVA) are presented in Table 8. Upon examination of the results, it was calculated that the total explanatory power (R-sq) of the model based on the 28-day UCS data is 94.10%, and the adjusted explanatory power is 77.87%. This indicates that a large portion of the experimental data can be explained. In terms of the significance of the factors, at a 5% significance level, the 6 mm HF additive (*p* = 0.031), SMS (%*p* = 0.033), and RHA (%*p* = 0.040) additives have a statistically significant effect. These results show that the short fiber additive is more effective than the long fiber additive, and that the appropriate amount of RHA and SMS additives significantly increases the strength of clay soils.

According to the analysis for the 28-day UCS obtained within the scope of the Taguchi experimental design, the optimum level determined for each factor was found to be 1.5% for the 6 mm HF additive, 1% for the 12 mm HF additive, 7.5% for the SMS, 7.5% for the RHA additive, and %OMC+10 for the optimum water content additive. The UCS value obtained from the laboratory experiments conducted according to the optimum design was found to be 1129 kPa, and the accuracy percentage of the design was calculated as 94.75%.

### 3.2. Freeze–Thaw (F-T) Resistance

**Figure 9:**
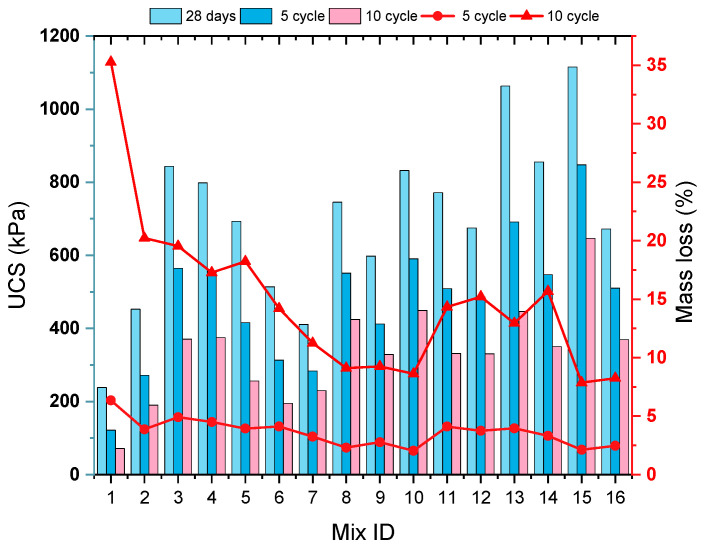
UCS values of mixtures after different F-T cycles.

Figure 9 presents a comparative distribution of the unconfined compressive strength (UCS) values measured after 28 days of curing and the UCS values obtained after 5 and 10 freeze–thaw (F-T) cycles. Overall, the graph demonstrates that F-T cycles reduced the mechanical strength in all samples. In particular, the application of 10 F-T cycles caused more significant strength losses compared to 5 cycles. After 5 F-T cycles, the UCS values of stabilized soil samples range approximately between 120 and 850 kPa, whereas after 10 F-T cycles, these values are observed to be between 70 and 650 kPa. The main reason for these outcomes is the presence of unreacted water in the structure of the mixtures. As is commonly known, water is a substance that expands when it freezes. The expansion of water within the specimens during freezing, subjected to F-T cycles, resulted in voids within their inner structure, thus forming weak structures. With an increasing number of F-T cycles, the microcracks that form in the soil structure reduce cohesive forces and internal friction, which leads to a decline in UCS values. In some mixtures (for example, Mix: 13 and 15), high strength levels were preserved even after 10 cycles, indicating the positive effect of these specific mixtures on F-T resistance. It is observed that, thanks to the hemp fiber (HF) addition and geopolymer formation in these mixtures, structural integrity was better maintained. Various studies in the literature have also found that the bearing capacity of soils decreases after FT cycles [62,63]. Another parameter examined after FT in the scope of the study is mass loss (%). Mass loss occurs due to the particles separated from the soil–geopolymer–fiber matrix and surface deteriorations and is mainly triggered by the expansion of ice crystals formed during freezing. When Figure 9 is examined, after 5 F-T cycles, generally low mass loss values between 2 and 6% were obtained in all mixtures. This indicates that the integrity of the matrix structure can be preserved mainly in the early cycles. After 10 F-T cycles, however, a noticeable increase in mass loss values (8–35%) was observed, but it was seen that the stabilized soil samples were successful in limiting this increase. This particularly emphasizes the importance of optimized geopolymer content and fiber combinations under long-term environmental effects.

**Figure 10:**
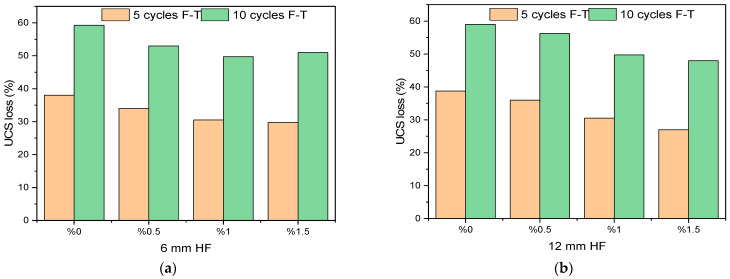

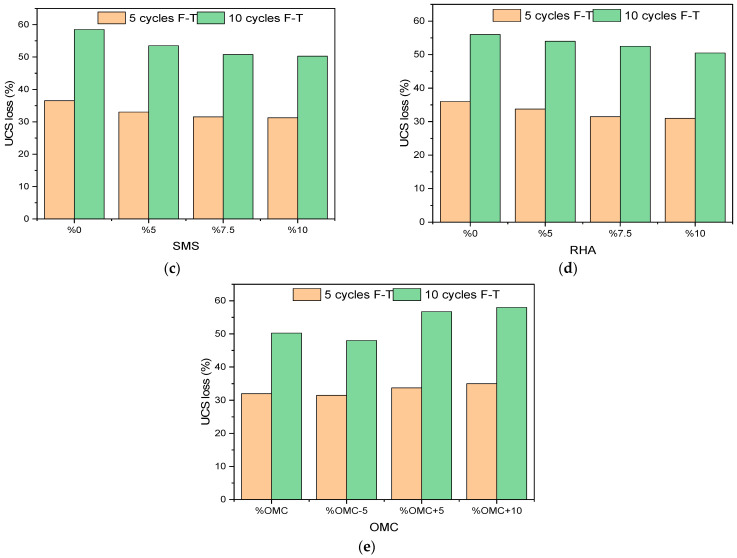
UCS loss (%) values of test samples after F-T cycles according to different parameters. (**a**) 6 mm HF effect; (**b**) 12 mm HF effect; (**c**) SMS effect; (**d**) RHA effect; (**e**) OMC effect.

According to the varying levels of each experimental parameter, the UCS loss obtained after 5 and 10 cycle freeze–thaw (F-T) cycles is presented in Figure 10.

#### 3.2.1. Effect of Hemp Fiber (HF) on UCS After F-T Cycles

As seen in Figure 10a, the samples containing 6 mm HF at a 0% ratio showed the highest strength loss after F-T cycles. In particular, after 10 F-T cycles, the strength loss reached approximately 60%. This shows that mixtures that are not reinforced with fiber are more prone to internal structural damage due to freezing and thawing. On the other hand, an increased fiber ratio (particularly at the 1.5% level) is accompanied by a significant reduction in loss of strength after both 5 and 10 freeze–thaw cycles. The addition of fibers aims to hinder the progression of microcracks developed within the matrix while allowing a more balanced distribution of internal forces caused by freezing and thawing. In this light, structural deterioration has significantly decreased [64,65,66]. As can be seen from the figure in Figure 10b, specimens with 12 mm HF at a 0% ratio showed a significant loss of strength after both 5-cycle and 10-cycle freeze–thaw operations, which is common with the results registered on stabilized soil specimens with 6 mm HF addition. An increase in the 12 mm fiber additive proportion resulted in a significant reduction in the loss of strength. In particular, a 1.5% additive ratio provided optimal performance with loss of strength reduced to 25% and 47% after 5 and 10 cycles, respectively. This implies that longer fiber lengths (12 mm) form better adhesion with the matrix, such that the propagation of cracks is hindered. It is therefore assumed that 12 mm fibers, due to longer bond length, are in a position to transmit pulling forces over a wider area, thus providing improved mechanical integrity under freeze–thaw impacts. In agreement with these research findings, Liu et al. also reported that longer fibers develop stronger fiber–matrix bond interfaces, such that a reduction in losses in strength is realized [67]. Several investigations revealed that natural as well as synthetic fibers exhibit reduced losses of strength as well as deformation when subjected to freeze–thaw impact, as is common with these test results [68,69]. Both 6 mm and 12 mm HF additives are therefore identified as efficient contributors to F-T resistance increase. The optimum additive proportion for both fiber lengths is therefore seen to be within 1–1.5%. In practical applications, the optimum fiber volume with respect to stabilizing soil is an important factor, with regard to which the fiber volume increase also needs to be assessed on workability considerations.

#### 3.2.2. Effect of Sodium Metasilicate (SMS) on UCS After F-T Cycles

The graph illustrated in Figure 10c reveals the substantial impacts of sodium metasilicate (SMS) incorporation on the reduction in strength following freeze–thaw (F-T) cycles. Samples lacking SMS (0% SMS) demonstrated considerable unconfined compressive strength (UCS) loss—37% after five cycles and 59% after ten cycles. The lack of SMS signifies inadequate activation of the precursor phase along with insufficient formation of geopolymer gel structures. Upon increasing the SMS incorporation rate to 5%, a reduction in strength loss was observed for both cycle counts, with additional decreases noted at 7.5% and 10% SMS concentrations. The minimal level of strength loss was recorded at 10% SMS incorporation. After ten cycles, samples with a 10% SMS addition exhibited a strength loss of 49%, representing approximately 10% less than samples without any additive. SMS facilitated the activation of precursors, namely rice husk ash (RHA) and partially kaolin clay (KC), aiding in the formation of N-A-S-H gels. The resultant gel structures enhanced the soil compactness and durability, consequently augmenting the soil resistance to freeze–thaw phenomena. Ahıskalı et al. indicated that employing SMS as an alkali activator fosters the development of binding gels (N-A-S-H) and that these constructs improve resistance to environmental effects, including freeze–thaw cycles [70]. Moreover, Jamalimoghadam et al. asserted that the inclusion of SMS diminishes soil permeability, curtails the occurrence of internal fissures, and thereby mitigates strength loss post-freeze–thaw cycles [71]. In summary, it can be concluded that the addition of SMS positively influences freeze–thaw resistance, with an optimal additive level approximating 10%. Nonetheless, it is essential to explore higher additive levels to ascertain the limits of effectiveness.

#### 3.2.3. Effect of Rice Husk Ash (RHA) on UCS After F-T Cycles

Figure 10d clearly illustrates the impact of rice husk ash (RHA) additive on soil strength following freeze–thaw cycles. The samples devoid of any additive (0% RHA) exhibited a 36% reduction in UCS following five cycles and a 56% reduction after ten cycles. The observed losses diminished progressively with an increase in RHA content. Specifically, with a 10% addition of RHA, the reduction in strength decreased to 30% after five cycles and to 51% after ten cycles. The beneficial impact of RHA is attributed to its significant content of amorphous silica, which interacts with the activator and water to generate binding phases. The gels generated from this reaction occupy the pores within the soil matrix, resulting in a more compact and resilient structure, thereby enhancing resistance to freeze–thaw cycles. Consistent with the existing literature, it has been observed that an increased precursor ratio improves the soil’s resistance to environmental factors, including freeze–thaw cycles [72,73].

#### 3.2.4. Effect of Optimum Moisture Content (OMC) on UCS After F-T Cycles

Figure 10e shows how adding varying quantities of water to kaolin clay samples affects their strength loss during freeze–thaw cycles. After five cycles, samples at the %OMC level lost around 32% of their strength, and after ten cycles, they lost about 50%. The post-F-T strength loss was the lowest in samples prepared at the %OMC−5 level, with losses of 30% after five cycles and 48% after 10 cycles. But at levels of %OMC+5 and %OMC+10, the strength reductions were more noticeable. At %OMC+10, losses were 35% after five cycles and 58% after ten cycles. The findings indicate that excess water adversely impacts the freeze–thaw strength of the soil. Excess water induces volumetric expansion during freezing within the pores. This generates microcracks and cavities in the internal structure, which make the material weaker. In the literature, it is noted that water ratios above the optimum moisture content cause a decrease in strength after F-T cycles, and that this is related to high porosity and increased ice lens formation [74,75]. Consequently, in terms of environmental durability, it is of critical importance to carefully determine and not exceed the optimum moisture content in order to improve the performance against freeze–thaw cycles.

#### 3.2.5. Statistical Analysis for UCS Loss After F-T According to Parameters

**Figure 11:**
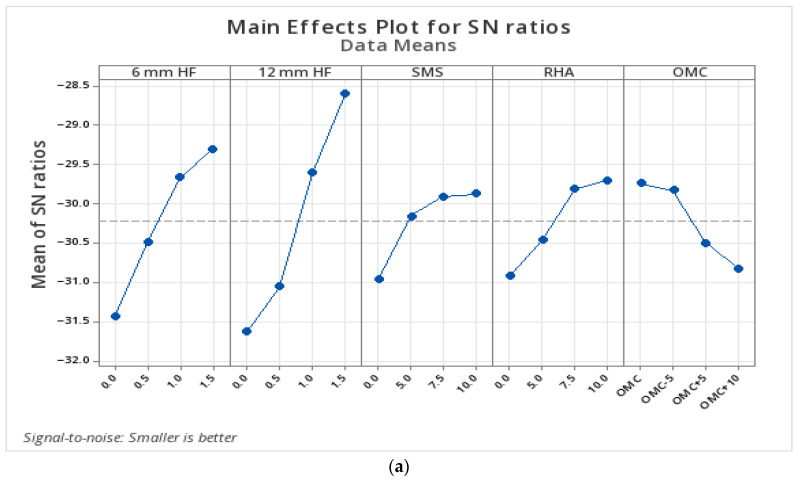

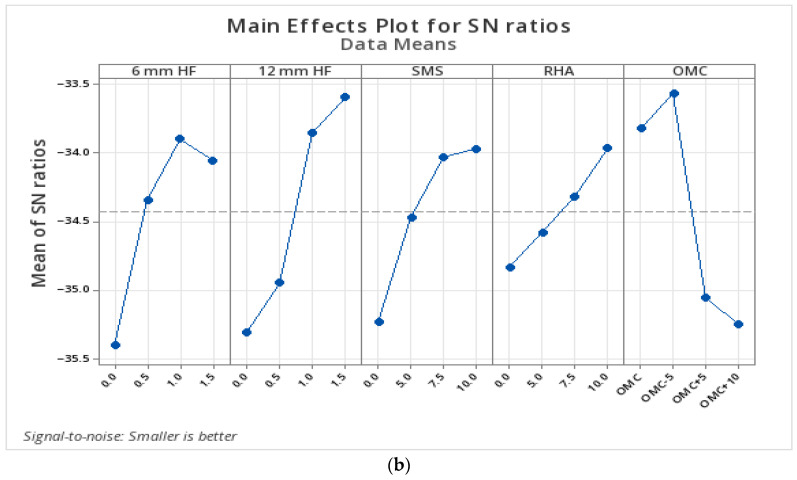
S/N chart of UCS loss after F-T. (**a**) 5 cycles; (**b**) 10 cycles.

For 5 and 10 F-T cycles, the average S/N ratio table (Table 9) obtained using the Taguchi method was used to statistically evaluate the effects of each factor level on UCS loss. This analysis was carried out using the “smaller is better” approach, as less strength loss is targeted after freeze–thaw cycles. In Figure 11, the main effect graph drawn according to the S/N ratios for 5 and 10 F-T cycles is presented.

After 5 and 10 F-T, it can be seen in Table 9 and Figure 11 that the most effective parameter is the addition of 12 mm hemp fiber (HF). As the fiber length increases and the ratio rises, the S/N ratio increases, meaning the loss of strength decreases. In both cycles, the addition of 6 mm HF also shows a similarly significant effect. Another noteworthy result here is that, after 10 F-T, OMC becomes the second most effective parameter. At the OMC+10 level, the lowest S/N ratio was measured. This indicates that excessive moisture weakens the microstructure of the matrix and, by increasing porosity, creates more ground for the expansion of water during freezing.

According to the ANOVA results presented in Table 10 for the freeze–thaw test conducted on the stabilized soil samples, all factors with a *p*-value < 0.05 were found to be statistically significant. For five F-T, the explanatory power of the model for the data is very high (R^2^ = 99.15%). As a result of the variance analysis of the strength loss data obtained after 10 cycles using the Taguchi method, the model’s R^2^ value was calculated as 99.21%, the Adj. R^2^ value is 97.05%, and the predicted value is 86.88%. These values indicate that the effects of the selected parameters on UCS loss are mostly explained and that the predictive strength of the model is high.

#### 3.2.6. Statistical Analysis for Mass Loss After F-T According to Parameters

**Figure 12:**
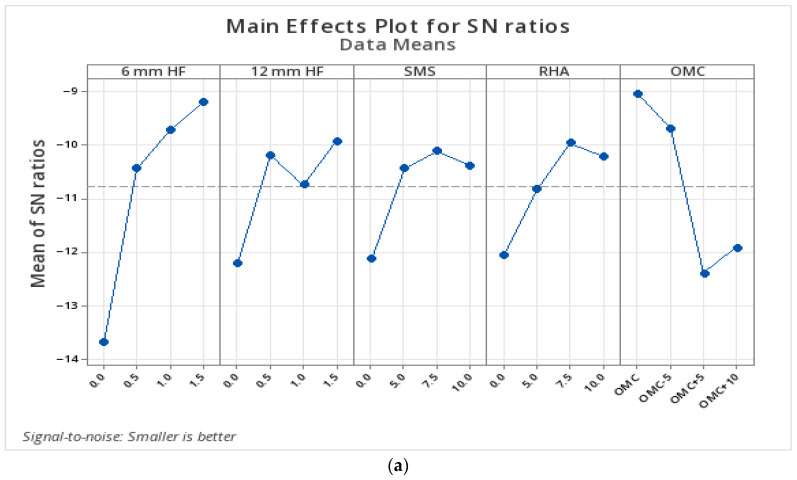

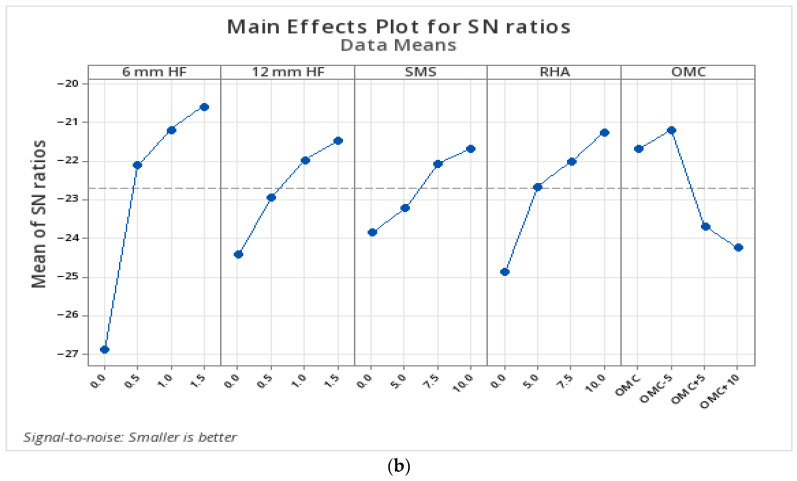
S/N chart of mass loss after F-T. (**a**) 5 cycles; (**b**) 10 cycles.

For 5 and 10 F-T cycles, the average S/N ratio table (Table 11) obtained using the Taguchi method was used to statistically evaluate the effects of each factor level on mass loss. This analysis was carried out using the “smaller is better” approach, as lower strength loss is targeted after freeze–thaw cycles. In Figure 12, the main effect graph drawn according to the S/N ratios for 5 and 10 F-T cycles is presented.

According to the Taguchi analysis, the parameter showing the highest effect for five F-T was the 6 mm HF ratio (Δ = 4.489). This was followed by OMC, 12 mm HF, RHA, and SMS, respectively. For 10 F-T, the most decisive parameter was the 6 mm fiber addition (Δ = 6.31, Rank 1). According to the S/N ratio, the lowest mass loss occurred in samples containing 1.5% fiber. This was followed by RHA (15.87% addition rate), OMC, and 12 mm HF, respectively. For both cycles, the fibers with a shorter length (6 mm HF) demonstrated a more effective structural integrity during freeze–thaw cycles. Looking at the results, it is clear that the mixes that did not have fiber and binder additions lost a lot of mass after 10 F-T. On the other hand, the mixes of fiber and geopolymer were able to stop mass loss by a large amount. For example, combination number 1 lost more than 35% of its mass, but mixes 15 and 16 lost less than 8% of their mass. Figure 12 shows that adding HF, SMS, and RHA to the stabilized soil samples made the S/N ratio less harmful, which means that the mass loss went down. In terms of OMC, the lowest mass loss occurred at the %OMC−5 and %OMC levels. At the %OMC+10 level, an increasing trend was observed, indicating that excessive moisture might increase structural disintegration due to freezing.

According to the ANOVA results presented in Table 12, after five F-T cycles, the 6 mm HF was the most dominant and significant parameter; in addition, the SMS and RHA additives also limited the mass loss. R^2^ = 96.06%, indicating a very high explanatory power. After 10 F-T cycles, 53.16% of the variance was explained by the 6 mm HF, and the overall model was very strong (R^2^ = 99.21%, *p* < 0.05). Furthermore, it was observed that the other parameters also made significant contributions. The model’s predictive power was also relatively high (R^2^pred = 86.88%).

### 3.3. Carbon Emission (CO_2_-e)

**Figure 13:**
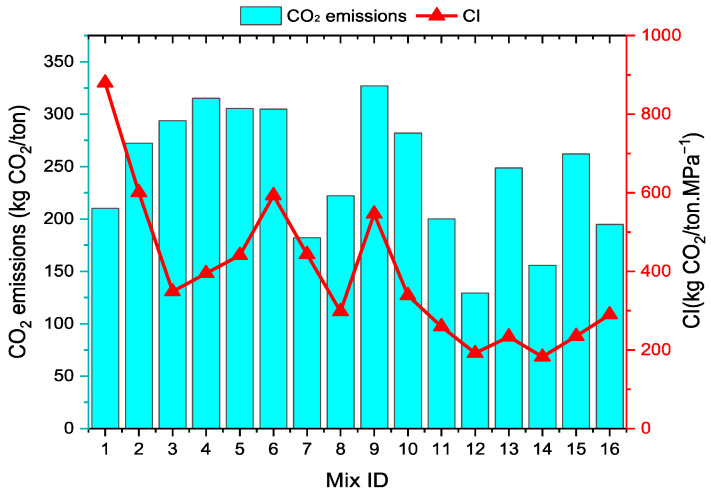
Carbon emission and carbon index of stabilized soil samples.

As shown in Figure 13, the carbon index CI (kgCO_2_/ton MPa^−1^) is also determined in order to assess the carbon emissions (kgCO_2_/ton) and environmental sustainability of the 16 stabilized soil mixtures. When the results are evaluated, it is observed that the total CO_2_-e values of the mixtures range between 129 and 326 kgCO_2_/ton. The CO_2_-e value of Mix 1, which does not contain HF or geopolymer and is considered the reference, is approximately 210 kgCO_2_/ton. This value arises from processes such as the transportation and placement of kaolin clay (KC) and water. The differences observed in other groups depend on the components that make up the mixtures. The main reason for the increase in the CO_2_-e value of stabilized soil samples is the sodium metasilicate (SMS) used as the geopolymer activator. As the proportion of SMS, which has a value of 0.45 kgCO_2_/kg, increases in the mixtures, the total CO_2_-e values of the mixtures also increase. The literature also reports that sodium-based activators significantly increase the carbon footprint [76,77]. Since RHA, another component used in stabilized soils, has a low emission value of 0.1 kgCO_2_/kg, its impact on the total CO_2_-e value is quite limited, which is an important advantage regarding the environmental friendliness of RHA. Another notable result regarding the total CO_2_-e values of the mixtures is that, as the volume of hemp fibers (HF) increases, the emission values decrease significantly. This is related to the carbon-negative property of HF, which is an organic component (−1.3 kgCO_2_/kg). For example, Mix 12, which has the lowest CO_2_-e value (129), has 2.5% HF. This mixture is about 39% lower than Mix 1. These findings indicate that employing HF for soil stabilization is a highly rational strategy for diminishing the carbon footprint. The CO_2_-e values of all combinations in this investigation are much lower than those of soils that have been stabilized with cement or lime in the literature. The primary explanation for this divergence is the usage of the geopolymer and HF in stabilization. Cement and lime, which are often used to stabilize soil, give off 0.95 and 0.77 kgCO_2_/kg, respectively. Zhang et al. found that the total CO_2_-e value of clay soil that was stabilized with 15% cement was about 638 kgCO_2_/ton [78]. In comparison, Zhu et al. calculated this value as approximately 465 kgCO_2_/ton in clay soil stabilized with 11% lime [79]. The combined effect of HF and geopolymer in soil stabilization is highly effective in reducing carbon emissions.

Another result presented in Figure 13 is the carbon index (CI). When the results are examined, the CI values range between 182 and 879 kgCO_2_/ton.MPa^−1^, depending on the composition of the mixtures. These results are based on the UCS and CO_2_-e values of the mixtures. In general, as the UCS value of the mixtures increases, the CI value generally decreases, and when it decreases, the opposite result occurs. For example, Mix 1, which has the lowest UCS value (0.24 MPa), has the highest CI value. The decrease in the CI value of the mixtures indicates an increase in sustainability. The CI value of Mix 8, which has a CO_2_-e value quite close to that of Mix 1, is about one-third less than that of Mix 1. The outcome here shows that, when determining the optimum soil stabilization mixture, not only should the UCS and total carbon emission values should be considered, but the carbon index should also be taken into account in terms of sustainability.

### 3.4. Microstructure Analysis

Within the scope of the study, a significant performance difference was observed between the 7th mixture, which exhibited the lowest strength value according to the 28-day UCS results of the test specimens, and the 15th mixture, which achieved the highest value. This variation can be explained by both macroscopic engineering parameters and microstructural bonding mechanisms, gel formation, and the distribution of vacancies in the matrix. In this regard, SEM images have facilitated the examination of microscopic structures influencing strength growth and have clarified the causes for the disparities among the mixtures.

**Figure 14:**
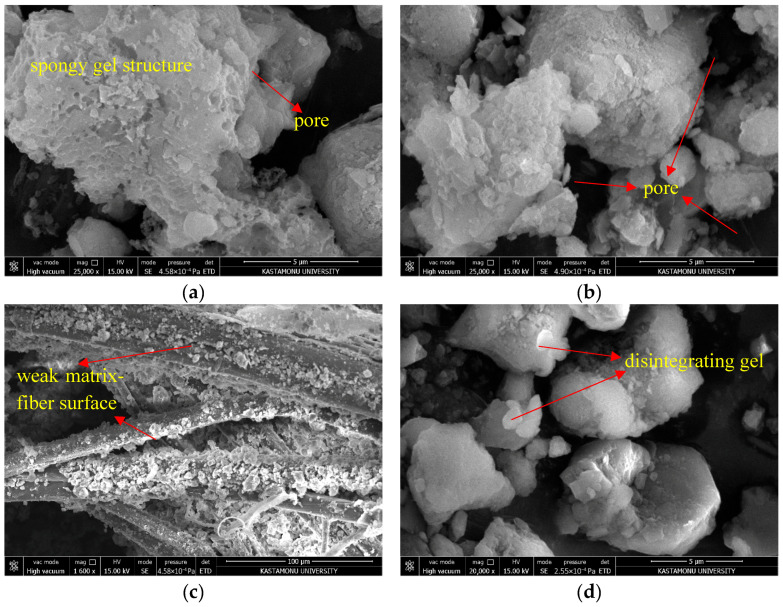
SEM images of Mixture 7: (**a**) matrix structure; (**b**) voids; (**c**) fiber–matrix interface; (**d**) after 10 F–T cycles.

Mixture 7 had 10% rice husk ash (RHA), 1.5% hemp fiber (HF), and 5% less water than the optimum level. In addition, this mixture did not include an activator, which is sodium metasilicate (SMS). Figure 14a shows that the microstructure of this mixture is not very ordered, has weak bonds, and has a very porous structure. RHA contains amorphous silica, but without an activator to add basic ions to the environment, aluminosilicates are very hard to dissolve. Because the dissolution is only partial, which is the first step in the geopolymerization process, the creation of a polymeric binder phase between the soil particles has been relatively limited. The SEM photos show that the structure is spongy and porous, since there is not much CSH and CH gels. These gels come from the CaO and SiO_2_ in RHA and kaolin clay (KC). The sharp unbound kaolin particles and the open voids between them, as seen in Figure 14b, clearly explain why the mechanical strength remains low. Furthermore, examining the SEM image presented in Figure 14c for the seventh mixture, the density of the matrix phase around the HF is quite limited. Not only has an adequate matrix phase failed to form on the surface of the fibers, but micro-voids and weak bond regions have also developed around the fibers. This indicates that the fiber does not contribute to stress transfer and acts merely as a passive filler. In Figure 14d, the SEM image of this mixture after 10 freeze–thaw (F-T) cycles is presented. The inter-particle bonds with low cohesion have weakened further due to ice expansion, resulting in the partial disintegration of the gel structures and the formation of large voids.

**Figure 15:**
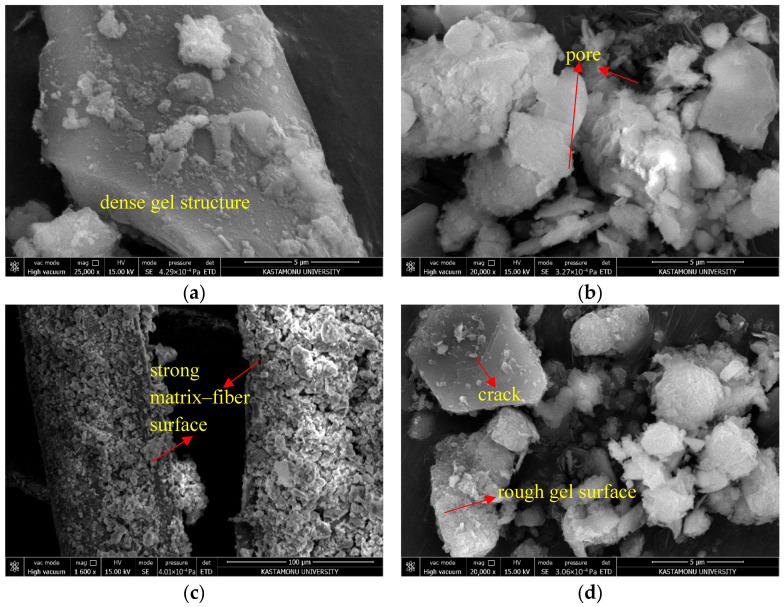
SEM images of Mixture 15: (**a**) matrix structure; (**b**) voids; (**c**) fiber–matrix interface; (**d**) after 10 F–T cycles.

SEM images of Mixture 15 are presented in Figure 15. This mixture was prepared with 5% RHA, 10% sodium metasilicate, 2.5% hemp fiber, and optimum water content. Figure 15a shows the SEM image of Mixture 15, which has a void-free and compact structure. This dense structure makes the combination less likely to be affected by mechanical forces than the weak structure shown in Mixture 7. The activator in this mixture made amorphous silica dissolve more easily in RHA, which made reactive ions develop. This immediately improved the density of the gel. The high water content also made it easier for ions to move around and speed up the reaction kinetics. The tight structure seen in Figure 15b shows that strong polymeric linkages were made between the soil particles and the amorphous gel phases (N-A-S-H and C-A-S-H types). The fact that this mixture contains fewer voids than Mixture 7 has had a good effect on the mechanical results. Figure 15c shows that the HF is integrated into the matrix both physically and mechanically in this mixture. SEM images indicate that the binder gel phases densely wrap the fiber surfaces, and microstructural continuity is maintained around the fibers as well. This suggests that the matrix forms strong adhesion with the fibers, thereby limiting crack propagation. Figure 15d presents the microstructure of Mixture 15 after 10 freeze–thaw (F-T) cycles. When the SEM image is examined, it is seen that the cracks within the gel structures remain limited in size. In addition, although there is slight roughness or layering marks on the gel surfaces, the structural integrity has been preserved. This result is related to the fact that the geopolymeric gel structures prevent capillary water movement and the displacement of dissolved ions caused by freeze–thaw cycles. There is a strong correlation between the mechanical response of the mixtures and the microstructural features observed in SEM analyses. Mixtures showing higher UCS exhibited denser gel networks, improved packed kaolinite, and stronger fiber–matrix interfaces. Conversely, low-strength mixtures displayed porous weakly bonded structures with insufficient gel formation. This confirms that geopolymer chemistry and fiber reinforcement mechanisms jointly govern the strength evolution.

As a result, the performance difference between the 7th and 15th mixtures can be directly explained at the microstructural level by the fundamental differences in their compositions. In the low-strength Mixture 7, the visible void areas occupy approximately 25–35% of the micrograph field, with interconnected pores typically ranging from 1 to 5 μm. In contrast, Mixture 15 shows a markedly denser matrix, where the apparent void area visually decreases to roughly 5–10%, and the pores are mostly isolated with estimated diameters below 0.5–1 μm. In addition, the fiber–matrix interface in Mixture 15 shows a nearly continuous gel coating, which reduces interfacial gaps by approximately 70–80% relative to Mixture 7. Such reductions in pore size, void fraction, and pore continuity are consistent with the improved UCS and the strongly reduced freeze–thaw strength losses of the optimized mixture. The absence of an activator, insufficient development of the binding phase, the weakness of the fiber–matrix interface, and inadequate water content constitute the main reasons for the low strength exhibited by the 7th mixture, whereas, in the 15th mixture, the optimal levels of these parameters have led to the development of high strength and environmental durability. In the literature, it has been emphasized that the mechanical and durability properties of geopolymers depend primarily on the materials in the mixture, the presence and type of fibers, and the curing conditions. Studies on geopolymer concrete or soil stabilization with geopolymers have highlighted that changes in the microstructure significantly alter the resistance of mixtures to mechanical and environmental effects. Within the scope of this study, it was found that changes in the mechanical properties and freeze–thaw resistance of the mixtures, depending on microstructural variations, parallel the results of previous studies [80,81,82]. However, the combined use of RHA and SMS in this research produced a denser and more continuous gel network than those reported in studies relying solely on NaOH activation. The synergy between HF reinforcement and geopolymer gel also resulted in a greater strength improvement than typical natural-fiber-stabilized clays. Moreover, the superior freeze–thaw durability observed in this work exceeds that of the cement- or lime-stabilized soils reported in the literature, mainly due to pore refinement and enhanced gel stability. Thus, the present study advances the existing research by demonstrating a more efficient coupling between fiber reinforcement and geopolymerization.

## 4. Conclusions

This work assessed the stabilization of kaolin clay (KC), characterized by its inferior engineering capabilities, using rice husk ash (RHA) and sodium metasilicate (SMS)-based geopolymer binder systems, focusing on mechanical and environmental performance. Furthermore, the impacts of incorporating hemp fiber (HF) and different water contents were methodically examined. The results derived from the parameters established using the Taguchi experimental design method are encapsulated below:

- The mixture with 5% RHA and 10% sodium metasilicate, which had an optimum water content, exhibited the highest unconfined compressive strength (UCS) value of all the mixtures.
- SEM analyses showed that the mixture formed a dense N-A-S-H gel and a compact microstructure, which makes it very strong. Conversely, mixes with diminished strength exhibited restricted binder structure formation and elevated porosity.
- The effects of hemp fiber reinforcement were different depending on the size and ratio of the fibers. Adding 1.5% hemp fibers that were 12 mm long to the mixture with the highest UCS value had a good effect.
- After the freeze–thaw cycles, mixtures without activators mostly lost their structural integrity. On the other hand, systems with RHA and sodium metasilicate kept most of their strength and showed a structure that was more resistant to environmental influences.
- The results show that geopolymer-based soil improvement systems have a lot of potential for engineering performance and environmental sustainability, since they make better use of natural resources and recycle industrial waste.
- This study was performed on a laboratory scale and did not assess varying environmental conditions, long-term durability performance, or behavior in field-scale applications. To immediately incorporate the findings into engineering design, future studies should examine sophisticated field tests and prolonged environmental exposure.
- From a sustainability perspective, this study aligns with several UN Sustainable Development Goals (SDGs), particularly SDG 9 (Industry, Innovation, and Infrastructure), SDG 11 (Sustainable Cities and Communities), and SDG 12 (Responsible Consumption and Production). By utilizing agricultural waste (RHA), a carbon-negative natural fiber (HF), and low-energy geopolymer technology, the proposed stabilization method significantly reduces carbon emissions compared with cement- and lime-based methods. The reduction in CI values further demonstrates the environmental efficiency of the developed system.

## Figures and Tables

**Table 1:** Index properties of KC.

Liquid limit LL (%)	42.94
Plastic limit PL (%)	26.40
Plasticity index PI (%)	16.54
Shrinkage limit (%)	12.27

**Table 2:** Chemical composition of RHA and KC (weight %).

Compound	RHA	KC
CaO	0.5	2.29
SiO_2_	86.2	62.02
Al_2_O_3_	0.6	20.34
Fe_2_O_3_	0.4	1.35
MgO	0.4	1.99
Na_2_O	0.4	0.13
SO_3_	0.3	0.23
K_2_O	1.9	0.17
LOI *	8.9	11.48

* Loss of ignition.

**Table 3:** Mechanical and physical properties of HF.

Fiber Length (mm)	Density (g/cm^3^)	Tensile Strength (MPa)	Elongation at Break (%)	Diameter (mm)
6–12	1.4	270–900 [27]	1–1.6 [28]	≅0.2

**Table 4:** Parameters and levels according to Taguchi experimental design.

Level	Parameter				
	6 mm HF (%)	12 mm HF (%)	RHA (%)	SMS (%)	OMC (%)
1	0	0	0	0	OMC
2	0.5	0.5	5	5	OMC−5
3	1	1	7.5	7.5	OMC+5
4	1.5	1.5	10	10	OMC+10

**Table 5:** Mixtures according to L16 orthogonal experimental design.

Mix No	6 mm HF (%)	12 mm HF (%)	RHA (%)	SMS (%)	OMC (%)
1	0	0	0	0	OMC
2	0	0.5	5	5	OMC−5
3	0	1	7.5	7.5	OMC+5
4	0	1.5	10	10	OMC+10
5	0.5	0	5	7.5	OMC+10
6	0.5	0.5	0	10	OMC+5
7	0.5	1	10	0	OMC−5
8	0.5	1.5	7.5	5	OMC
9	1	0	7.5	10	OMC−5
10	1	0.5	10	7.5	OMC
11	1	1	0	5	OMC+10
12	1	1.5	5	0	OMC+5
13	1.5	0	10	5	OMC+5
14	1.5	0.5	7.5	0	OMC+10
15	1.5	1	5	10	OMC
16	1.5	1.5	0	7.5	OMC−5

**Table 6:** CO_2_ emissions value of materials in test samples.

Material	CO_2_-e (kg-CO_2_/kg)	Reference
KC	0.07	[36]
RHA	0.10	[37]
SMS	0.45	[38]
HF	−1.29	[39]
Water	0.0003	[40]

**Table 7:** The effects of the factors at 28-day UCS results.

Level	6 mm HF	12 mm HF	SMS	RHA	OMC
1	54.31	55.11	54.02	53.77	56.09
2	55.19	56.10	56.87	57.21	54.37
3	57.07	57.37	57.53	57.57	57.46
4	59.17	57.15	57.31	57.18	57.81
Delta	4.85	2.26	3.51	3.80	3.44
Rank	1	5	3	2	4

**Table 8:** Results of the analysis of variance (ANOVA) for 28-day USC test.

Source	DF	Seq SS	Contribution	Adj SS	Adj MS	F-Value	*p*-Value
SMS	1	118,641	15.10%	118,641	118,641	10.23	0.033
RHA	1	104,313	13.28%	104,313	104,313	9.00	0.040
6 mm HF	3	308,094	39.21%	308,094	102,698	8.86	0.031
12 mm HF	3	46,596	5.93%	46,596	15,532	1.34	0.380
OMC	3	161,687	20.58%	161,687	53,896	4.65	0.086
Error	4	46,369	5.90%	46,369	11,592		
Total	15	785,700	100.00%				

**Table 9:** The effect of factors on UCS loss after F-T.

5 F-T	Level	6 mm HF	12 mm HF	SMS	RHA	OMC
	1	−31.44	−31.64	−30.96	−30.93	−29.74
	2	−30.50	−31.06	−30.16	−30.46	−29.82
	3	−29.66	−29.61	−29.91	−29.82	−30.50
	4	−29.30	−28.60	−29.87	−29.70	−30.83
	Delta	2.14	3.04	1.09	1.23	1.10
	Rank	2	1	5	3	4
10 F-T	Level	6 mm HF	12 mm HF	SMS	RHA	OMC
	1	−35.40	−35.31	−35.23	−34.83	−33.82
	2	−34.34	−34.95	−34.47	−34.58	−33.57
	3	−33.90	−33.85	−34.03	−34.32	−35.06
	4	−34.05	−33.59	−33.97	−33.97	−35.25
	Delta	1.51	1.71	1.26	0.87	1.69
	Rank	3	1	4	5	2

**Table 10:** Results of the analysis of variance (ANOVA) UCS loss after F-T.

5 F-T	Source	DF	Seq SS	Contribution	Adj SS	Adj MS	F-Value	*p*-Value
	SMS	1	66.516	9.88%	66.516	66.516	46.33	0.002
	RHA	1	61.116	9.08%	61.116	61.116	42.57	0.003
	6 mm HF	3	171.188	25.44%	171.188	57.063	39.75	0.002
	12 mm HF	3	337.188	50.11%	337.188	112.396	78.29	0.001
	OMC	3	31.187	4.63%	31.187	10.396	7.24	0.043
	Error	4	5.743	0.85%	5.743	1.436		
	Total	15	672.938	100.00%				
10 F-T	Source	DF	Seq SS	Contribution	Adj SS	Adj MS	F-Value	*p*-Value
	SMS	1	165.03	15.52%	165.029	165.029	78.99	0.001
	RHA	1	63.11	5.94%	63.114	63.114	30.21	0.005
	6 mm HF	3	213.50	20.08%	213.500	71.167	34.06	0.003
	12 mm HF	3	327.50	30.81%	327.500	109.167	52.25	0.001
	OMC	3	285.50	26.86%	285.500	95.167	45.55	0.002
	Error	4	8.36	0.79%	8.357	2.089		
	Total	15	1063.00	100.00%				

**Table 11:** The effect of factors on mass loss after F-T.

5 F-T	Level	6 mm HF	12 mm HF	SMS	RHA	OMC
	1	−13.689	−12.200	−12.121	−12.049	−9.033
	2	−10.429	−10.183	−10.434	−10.820	−9.687
	3	−9.718	−10.730	−10.104	−9.960	−12.393
	4	−9.200	−9.923	−10.378	−10.207	−11.923
	Delta	4.489	2.277	2.017	2.090	3.360
	Rank	1	3	5	4	2
10 F-T	Level	6 mm HF	12 mm HF	SMS	RHA	OMC
	1	−26.91	−24.43	−23.86	−24.88	−21.69
	2	−22.12	−22.95	−23.22	−22.67	−21.20
	3	−21.20	−21.97	−22.06	−22.02	−23.69
	4	−20.60	−21.48	−21.68	−21.26	−24.25
	Delta	6.31	2.95	2.18	3.62	3.05
	Rank	1	4	5	2	3

**Table 12:** Results of the analysis of variance (ANOVA) mass loss after F-T.

5 F-T	Source	DF	Seq SS	Contribution	Adj SS	Adj MS	F-Value	*p*-Value
	SMS	1	1.6907	8.72%	1.6907	1.6907	8.87	0.041
	RHA	1	1.5404	7.95%	1.5404	1.5404	8.08	0.047
	6 mm HF	3	9.3837	48.41%	9.3837	3.1279	16.40	0.010
	12 mm HF	3	2.4530	12.66%	2.4530	0.8177	4.29	0.097
	OMC	3	3.5528	18.33%	3.5528	1.1843	6.21	0.055
	Error	4	0.7628	3.94%	0.7628	0.1907		
	Total	15	19.3834	100.00%				
10 F-T	Source	DF	Seq SS	Contribution	Adj SS	Adj MS	F-Value	*p*-Value
	SMS	1	70.468	10.09%	70.468	70.468	36.18	0.004
	RHA	1	110.761	15.87%	110.761	110.761	56.86	0.002
	6 mm HF	3	371.127	53.16%	371.127	123.709	63.51	0.001
	12 mm HF	3	99.481	14.25%	99.481	33.160	17.02	0.010
	OMC	3	38.482	5.51%	38.482	12.827	6.59	0.050
	Error	4	7.791	1.12%	7.791	1.948		
	Total	15	698.110	100.00%				

## Data Availability

The original contributions presented in this study are included in the article. Further inquiries can be directed to the corresponding author.

## References

[1] Afrin H. (2017). A review on different types soil stabilization techniques. Int. J. Transp. Eng. Technol..

[2] Firoozi A.A., Guney Olgun C., Firoozi A.A., Baghini M.S. (2017). Fundamentals of soil stabilization. Int. J. Geo-Eng..

[3] Barcelo L., Kline J., Walenta G., Gartner E. (2014). Cement and carbon emissions. Mater. Struct..

[4] Xu Z., Xie X., He M., Luo Z., Wu J., Bin J., Yang L., Zhang B. (2025). Research Review of Reaction Mechanism and Mechanical Properties of Chemically Solidified Silt. Buildings.

[5] Cong P., Cheng Y. (2021). Advances in geopolymer materials: A comprehensive review. J. Traffic Transp. Eng. (Engl. Ed.).

[6] Nodehi M., Taghvaee V.M. (2022). Alkali-activated materials and geopolymer: A review of common precursors and activators addressing circular economy. Circ. Econ. Sustain..

[7] Chen X., Sutrisno A., Struble L.J. (2018). Effects of calcium on setting mechanism of metakaolin-based geopolymer. J. Am. Ceram. Soc..

[8] Ma C.-K., Awang A.Z., Omar W. (2018). Structural and material performance of geopolymer concrete: A review. Constr. Build. Mater..

[9] Wong L.S. (2022). Durability performance of geopolymer concrete: A review. Polymers.

[10] Abbas R., Khereby M.A., Ghorab H.Y., Elkhoshkhany N. (2020). Preparation of geopolymer concrete using Egyptian kaolin clay and the study of its environmental effects and economic cost. Clean Technol. Environ. Policy.

[11] Almalkawi A.T., Balchandra A., Soroushian P. (2019). Potential of using industrial wastes for production of geopolymer binder as green construction materials. Constr. Build. Mater..

[12] Anburuvel A. (2023). The role of activators in geopolymer-based stabilization for road construction: A state-of-the-art review. Multiscale Multidiscip. Model. Exp. Des..

[13] Murmu A.L., Patel A. (2020). Studies on the properties of fly ash–rice husk ash-based geopolymer for use in black cotton soils. Int. J. Geosynth. Ground Eng..

[14] Tesanasin T., Suksiripattanapong C., Kuasakul T., Thongkhwan T., Tabyang W., Thumrongvut J., Keawsawasvong S. (2024). Comparison between cement-rice husk ash and cement-rice husk ash one-part geopolymer for stabilized soft clay as deep mixing material. Transp. Infrastruct. Geotechnol..

[15] Wang S., Xue Q., Ma W., Zhao K., Wu Z. (2021). Experimental study on mechanical properties of fiber-reinforced and geopolymer-stabilized clay soil. Constr. Build. Mater..

[16] Medina-Martinez C.J., Sandoval-Herazo L.C., Zamora-Castro S.A., Vivar-Ocampo R., Reyes-Gonzalez D. (2022). Natural fibers: An alternative for the reinforcement of expansive soils. Sustainability.

[17] Shalchian M.M., Arabani M. (2022). A review of soil reinforcement with planetary fibers. J. Soil Sci. Plant Nutr..

[18] Sharma V., Vinayak H.K., Marwaha B.M. (2015). Enhancing compressive strength of soil using natural fibers. Constr. Build. Mater..

[19] Barbhuiya S., Das B.B. (2022). A comprehensive review on the use of hemp in concrete. Constr. Build. Mater..

[20] Syed M., GuhaRay A., Goel D., Asati K., Peng L. (2020). Effect of freeze–thaw cycles on black cotton soil reinforced with coir and hemp fibres in alkali-activated binder. Int. J. Geosynth. Ground Eng..

[21] Vafaei A., Choobbasti A.J., Koutenaei R.Y., Vafaei A., Afrakoti M.P., Kutanaei S.S. (2022). Experimental investigation of the mechanical behavior and engineering properties of sand reinforced with hemp fiber. Arab. J. Geosci..

[22] (2025). Standard Practice for Classification of Soils for Engineering Purposes (Unified Soil Classification System).

[23] Hossain S.S., Roy P., Bae C.-J. (2021). Utilization of waste rice husk ash for sustainable geopolymer: A review. Constr. Build. Mater..

[24] Muñoz Pérez S.P., Charca Mamani S., Villena Zapata L.I., Piedra J.L.L., Gonzales Ayasta S., Rodriguez Lafitte E.D., Aparicio Roque F.G., Coronado Zuloeta O. (2023). Influence of rice husk ash (RHA) with gypsum and ichu fibers in the processing of geopolymers. Innov. Infrastruct. Solut..

[25] Matsimbe J., Dinka M., Olukanni D., Musonda I. (2022). Geopolymer: A systematic review of methodologies. Materials.

[26] Yang J., Bai H., He X., Zeng J., Su Y., Wang X., Zhao H., Mao C. (2023). Performances and microstructure of one-part fly ash geopolymer activated by calcium carbide slag and sodium metasilicate powder. Constr. Build. Mater..

[27] Bogoeva-Gaceva G., Avella M., Malinconico M., Buzarovska A., Grozdanov A., Gentile G., Errico M. (2007). Natural fiber eco-composites. Polym. Compos..

[28] Mueller D.H., Krobjilowski A. (2003). New discovery in the properties of composites reinforced with natural fibers. J. Ind. Text..

[29] Basha E., Hashim R., Mahmud H., Muntohar A. (2005). Stabilization of residual soil with rice husk ash and cement. Constr. Build. Mater..

[30] Alcan H.G., Bayrak B., Öz A., Kavaz E., Kaplan G., Çelebi O., Aydın A.C. (2023). A comprehensive characterization on geopolymer concretes with low content slag and quartz aggregates: The shielding features. Radiat. Eff. Defects Solids.

[31] Patil A.A., Chore H., Dode P. (2014). Effect of curing condition on strength of geopolymer concrete. Adv. Concr. Constr..

[32] Sajan P., Jiang T., Lau C., Tan G., Ng K. (2021). Combined effect of curing temperature, curing period and alkaline concentration on the mechanical properties of fly ash-based geopolymer. Clean. Mater..

[33] (2018). Standard Test Methods for Compressive Strength of Molded Soil-Cement Cylinders.

[34] (2012). Standard Test Methods for Freezing and Thawing Compacted Soil-Cement Mixtures.

[35] Shi Y., Long G., Ma C., Xie Y., He J. (2019). Design and preparation of ultra-high performance concrete with low environmental impact. J. Clean. Prod..

[36] Zhou D., Wang R., Tyrer M., Wong H., Cheeseman C. (2017). Sustainable infrastructure development through use of calcined excavated waste clay as a supplementary cementitious material. J. Clean. Prod..

[37] Ozturk E., Ince C., Derogar S., Ball R. (2022). Factors affecting the CO_2_ emissions, cost efficiency and eco-strength efficiency of concrete containing rice husk ash: A database study. Constr. Build. Mater..

[38] McLellan B.C., Williams R.P., Lay J., Van Riessen A., Corder G.D. (2011). Costs and carbon emissions for geopolymer pastes in comparison to ordinary portland cement. J. Clean. Prod..

[39] Scrucca F., Ingrao C., Maalouf C., Moussa T., Polidori G., Messineo A., Arcidiacono C., Asdrubali F. (2020). Energy and carbon footprint assessment of production of hemp hurds for application in buildings. Environ. Impact Assess. Rev..

[40] Ma C., Zhao B., Wang L., Long G., Xie Y. (2020). Clean and low-alkalinity one-part geopolymeric cement: Effects of sodium sulfate on microstructure and properties. J. Clean. Prod..

[41] Jafari M.M., Bagheripour M.H., Yaghoubi E., Abolghasemi Mahani A. (2025). Impact of Curing Age and Capillary Action on Microstructural and Stress–Strain Response of a Geopolymer-Stabilized Sandy Soil. J. Mater. Civ. Eng..

[42] Wassie T.A., Demir G., Köktan U. (2023). Influence of Curing Time and Initial Moisture Content on Metakaolin-Based Geopolymer-Stabilized Soft Soil. Adv. Civ. Eng..

[43] Kannan G., Sujatha E.R. (2023). Effect of nano additive on mechanical properties of natural fiber reinforced soil. J. Nat. Fibers.

[44] Yixian W., Panpan G., Shengbiao S., Haiping Y., Binxiang Y. (2016). Study on strength influence mechanism of fiber-reinforced expansive soil using jute. Geotech. Geol. Eng..

[45] Khalil H.A., Hossain M.S., Rosamah E., Azli N., Saddon N., Davoudpoura Y., Islam M.N., Dungani R. (2015). The role of soil properties and it’s interaction towards quality plant fiber: A review. Renew. Sustain. Energy Rev..

[46] Muntohar A.S., Widianti A., Hartono E., Diana W. (2013). Engineering properties of silty soil stabilized with lime and rice husk ash and reinforced with waste plastic fiber. J. Mater. Civ. Eng..

[47] Wei L., Chai S.X., Zhang H.Y., Shi Q. (2018). Mechanical properties of soil reinforced with both lime and four kinds of fiber. Constr. Build. Mater..

[48] Zhang H.-Y., Liu J.-C., Wu B. (2021). Mechanical properties and reaction mechanism of one-part geopolymer mortars. Constr. Build. Mater..

[49] Nath P., Sarker P.K. (2015). Use of OPC to improve setting and early strength properties of low calcium fly ash geopolymer concrete cured at room temperature. Cem. Concr. Compos..

[50] Xu H., Van Deventer J. (2000). The geopolymerisation of alumino-silicate minerals. Int. J. Miner. Process..

[51] Youssef N., Rabenantoandro A.Z., Lafhaj Z., Dakhli Z., Hage Chehade F., Ducoulombier L. (2021). A novel approach of geopolymer formulation based on clay for additive manufacturing. Constr. Robot..

[52] Ghosh A., Subbarao C. (2007). Strength characteristics of class F fly ash modified with lime and gypsum. J. Geotech. Geoenviron. Eng..

[53] Umar I.H., Lin H., Ibrahim A.S. (2023). Laboratory testing and analysis of clay soil stabilization using waste marble powder. Appl. Sci..

[54] Yadav J.S., Tiwari S.K. (2016). Behaviour of cement stabilized treated coir fibre-reinforced clay-pond ash mixtures. J. Build. Eng..

[55] Antiohos S., Papadakis V., Tsimas S. (2014). Rice husk ash (RHA) effectiveness in cement and concrete as a function of reactive silica and fineness. Cem. Concr. Res..

[56] Khale D., Chaudhary R. (2007). Mechanism of geopolymerization and factors influencing its development: A review. J. Mater. Sci..

[57] Párraga Morales D., Rivera E.O., Lotero A., Moncaleano C.J., Consoli N.C. (2024). Potential reuse of Andean highlands Tin tailings in geotechnical works through geopolymer binder stabilization. Geotech. Geol. Eng..

[58] Embong R., Kusbiantoro A., Shafiq N., Nuruddin M.F. (2016). Strength and microstructural properties of fly ash based geopolymer concrete containing high-calcium and water-absorptive aggregate. J. Clean. Prod..

[59] Abdila S.R., Abdullah M.M.A.B., Ahmad R., Burduhos Nergis D.D., Rahim S.Z.A., Omar M.F., Sandu A.V., Vizureanu P., Syafwandi (2022). Potential of soil stabilization using ground granulated blast furnace slag (GGBFS) and fly ash via geopolymerization method: A review. Materials.

[60] Horpibulsuk S., Rachan R., Raksachon Y. (2009). Role of fly ash on strength and microstructure development in blended cement stabilized silty clay. Soils Found..

[61] Zhang D.-W., Zhao K.-F., Xie F.-z., Li H., Wang D.-m. (2020). Effect of water-binding ability of amorphous gel on the rheology of geopolymer fresh pastes with the different NaOH content at the early age. Constr. Build. Mater..

[62] de Jesús Arrieta Baldovino J., dos Santos Izzo R.L., Rose J.L. (2021). Effects of freeze–thaw cycles and porosity/cement index on durability, strength and capillary rise of a stabilized silty soil under optimal compaction conditions. Geotech. Geol. Eng..

[63] Nguyen T.T.H., Cui Y.-J., Ferber V., Herrier G., Ozturk T., Plier F., Puiatti D., Salager S., Tang A.M. (2019). Effect of freeze-thaw cycles on mechanical strength of lime-treated fine-grained soils. Transp. Geotech..

[64] Farhan K.Z., Megat A.M.J., Demirboğa R. (2023). Performance of polypropylene fiber reinforced GGBFS-based alkali activated composites under sulfate and freeze–thaw conditions. Mater. Struct..

[65] Li Y., Zhang Q., Wang R., Xiong X., Li Y., Wang J. (2022). Experimental investigation on the dynamic mechanical properties and microstructure deterioration of steel fiber reinforced concrete subjected to freeze–thaw cycles. Buildings.

[66] Zhang P., Shi B., Dai X., Chen C., Lai C. (2025). A State-of-the-Art Review on the Freeze–Thaw Resistance of Sustainable Geopolymer Gel Composites: Mechanisms, Determinants, and Models. Gels.

[67] Liu Y., Li H., Feng Z., Ge L., Li R., Liu S. (2024). Study on the interfacial bonding properties between alkali-treated bamboo fibers and high-performance seawater sea-sand concrete. Constr. Build. Mater..

[68] Boz A., Sezer A. (2018). Influence of fiber type and content on freeze-thaw resistance of fiber reinforced lime stabilized clay. Cold Reg. Sci. Technol..

[69] Kravchenko E., Liu J., Niu W., Zhang S. (2018). Performance of clay soil reinforced with fibers subjected to freeze-thaw cycles. Cold Reg. Sci. Technol..

[70] Ahıskalı A., Bayrak B., Toklu K., Bayraktar O.Y., Kaplan G., Aydın A.C. (2025). Characterizing the Chemistry of One-Part Green Geopolymer Foams: The Role of Silica Fume and Fiber Hybridization. ACS Omega.

[71] Jamalimoghadam M., Bahmyari H. (2023). Freeze–Thaw Characteristics of Slaking Marl Clay Stabilized with a Binder Based on Alkali-Activated Recycled Glass Powder. J. Mater. Civ. Eng..

[72] Li B., Luo F., Li X., Liu J. (2024). Mechanical properties evolution of clays treated with rice husk ash subjected to freezing-thawing cycles. Case Stud. Constr. Mater..

[73] Olgun M. (2013). The effects and optimization of additives for expansive clays under freeze–thaw conditions. Cold Reg. Sci. Technol..

[74] Huang S., He Y., Yu S., Cai C. (2022). Experimental investigation and prediction model for UCS loss of unsaturated sandstones under freeze-thaw action. Int. J. Min. Sci. Technol..

[75] Qiu E., He Q., Chen Q., Sun X., Zhang R., Qu M., Wan X. (2023). Influence of freeze–thaw cycles on mechanical properties of moraine soils. Transp. Geotech..

[76] Tan J., Cai J., Li X., Pan J., Li J. (2020). Development of eco-friendly geopolymers with ground mixed recycled aggregates and slag. J. Clean. Prod..

[77] Zannerni G.M., Fattah K.P., Al-Tamimi A.K. (2020). Ambient-cured geopolymer concrete with single alkali activator. Sustain. Mater. Technol..

[78] Zhang T., Yue X., Deng Y., Zhang D., Liu S. (2014). Mechanical behaviour and micro-structure of cement-stabilised marine clay with a metakaolin agent. Constr. Build. Mater..

[79] Zhu F., Li Z., Dong W., Ou Y. (2019). Geotechnical properties and microstructure of lime-stabilized silt clay. Bull. Eng. Geol. Environ..

[80] Alcan H.G. (2025). Mechanical, Durability, and Environmental Impact Properties of Natural and Recycled Fiber Geopolymer with Zero Waste Approach: Alternative to Traditional Building Materials. Polymers.

[81] Odeh N.A., Al-Rkaby A.H. (2022). Strength, Durability, and Microstructures characterization of sustainable geopolymer improved clayey soil. Case Stud. Constr. Mater..

[82] Yılmazoglu M.U. (2025). Alkali-Activated Stabilization of Silt Soil Using Garlic Husk Ash: Mechanical, Microstructural, and Durability Performance. Appl. Sci..

